# Acral skin vasoreactivity and thermosensitivity to hand cooling following 5 days of intermittent whole body cold exposure

**DOI:** 10.1152/ajpregu.00021.2022

**Published:** 2022-05-03

**Authors:** Michail E. Keramidas, Roger Kölegård, Pit Gäng, Frederick Wilkins, Antonis Elia, Ola Eiken

**Affiliations:** Division of Environmental Physiology, Swedish Aerospace Physiology Center, KTH Royal Institute of Technology, Stockholm, Sweden

**Keywords:** CIVD, cold adaptation, cold injury, cutaneous circulation, habituation

## Abstract

We sought to examine whether short-term, whole body cold acclimation would modulate finger vasoreactivity and thermosensitivity to localized cooling. Fourteen men were equally assigned to either the experimental (CA) or the control (CON) group. The CA group was immersed to the chest in 14°C water for ≤120 min daily over a 5-day period while the skin temperature of the right-hand fingers was clamped at ∼35.5°C. The CON group was instructed to avoid any cold exposure during this period. Before and after the intervention, both groups performed, on two different consecutive days, a local cold provocation trial consisting of a 30-min hand immersion in 8°C water while immersed to the chest once in 21°C (mild-hypothermic trial; 0.5°C fall in rectal temperature from individual preimmersion values) and on the other occasion in 35.5°C (normothermic trial). In the CA group, the cold-induced reduction in finger temperature was less (mild-hypothermic trial: *P* = 0.05; normothermic trial: *P* = 0.02), and the incidence of the cold-induced vasodilation episodes was greater (in normothermic trials: *P* = 0.04) in the post- than in the preacclimation trials. The right-hand thermal discomfort was also attenuated (mild-hypothermic trial: *P* = 0.04; normothermic trial: *P* = 0.01). The finger temperature responses of the CON group did not vary between testing periods. Our findings suggest that repetitive whole body exposure to severe cold within a week may attenuate finger vasoreactivity and thermosensitivity to localized cooling. These regional thermo-adaptions were ascribed to central neural habituation produced by the iterative, generalized cold stimulation.

## INTRODUCTION

During exposure to cold, a rapid sympathetically mediated increase in cutaneous vasomotion occurs, limiting heat loss to the surroundings. In acral skin regions, however, the overall drive for vasoconstriction is often interrupted by transient elevations in blood flow and temperature [cold-induced vasodilation (CIVD)] (1), which alleviate regional thermal pain and discomfort (2), and perhaps enhance resistance against freezing cold injury (3, 4). Although the exact mechanisms underlying the CIVD response are still unsettled (see Ref. 5), recurrent evidence suggests that the incidence and magnitude of the response are mediated, to a degree, centrally, via withdrawal of sympathetic tone (6, 7).

Cross-sectional, population-based studies have indicated that long-term exposure to cold may lead to peripheral thermal adaptations, conceivably reducing susceptibility to local cold injury. For instance, Arctic natives (e.g., Inuit and Saami), as well as workers who repeatedly and chronically are exposed to low ambient temperatures (e.g., fish filleters) appear to have enhanced hand blood flow and temperature, and to perceive less thermal discomfort during cold provocation (8–16). Yet, whether these microvascular and thermoperceptual adjustments describe an inherited (genotypic) or acquired (phenotypic) feature remains unknown. Furthermore, in such cross-sectional studies, neither the cumulative adaptation (thermal) impulse can be quantified nor can the relative contribution be determined of thermal input from the body core and shell (i.e., whole body, and/or local skin cooling) respectively, in the adaptation process.

Considering the methodological caveats of the aforementioned experimental models, most of the laboratory investigations have sought to examine, in nonacclimatized individuals, the impact of long-term, repeated local appendage (i.e., whole limb, or single digit) cooling on the acral-skin vasomotor function, and particularly on CIVD. The findings derived from local cold-acclimation studies, however, are equivocal: a few have observed an enhancement of the CIVD response (17–19), whereas others have detected either a reduction (20–22), or no change (23–25).

Notably, information pertaining to the effects of whole body cold acclimation on the acral-skin thermoregulatory capacity is relatively scarce. O’Brien et al. (26) and Wakabayashi et al. (27) found that finger vasoreactivity to localized cooling was augmented (i.e., the vasoconstrictor and CIVD responses were increased and decreased, respectively) after 5 and 4 wk, respectively, of repetitive deep body cooling, provoked by whole body immersion in cold water. Both studies, however, utilized acclimation protocols of relatively long duration, leading to the induction of systemic “insulative” adaptation, which intrinsically is described by enhanced sympathetic constrictor responsiveness to cold. The type of cold adaptation developed is determined, not only by the severity of the stressor used, but also by the duration of the intervention (28, 29). Thus, it has been postulated that the insulative adaptation is probably preceded by a “habituation” phase (or hypothermic adaptation) (13, 30), during which, along with downregulation of shivering thermogenesis, the elevations in arterial pressure and peripheral vasomotion in response to a whole body cold stimulus are typically blunted, and the perceived thermal discomfort is attenuated (for review, see Ref. 31).

The present study, therefore, aimed to examine whether, and to what extent, central habituation ensued from the iterative application of a fixed whole body cold stimulus would modulate finger vasoreactivity and thermosensitivity to localized cooling. For this purpose, we monitored thermal, circulatory, and perceptual responses during a hand cold (8°C) provocation trial, before and after a 5-day cold-acclimation regimen. Considering that the whole body thermal state dictates the magnitude of cutaneous vasoreactivity to localized cold stress (6, 32–34), finger responsiveness was evaluated while subjects were rendered once mildly hypothermic and on the other occasion normothermic. Also, to isolate any confounding influence derived from local thermal stress during the adaptation process, a closed-loop acclimation protocol was used, during which individuals’ body core and skin temperature were manipulated daily by means of immersion to the chest in 14°C water for a maximum 2-h period, whereas the skin-surface temperature of the region of interest (i.e., the right-hand fingers) was clamped at 35.5°C. We hypothesized that the cold acclimation regimen would blunt the magnitude of sympathetic response instigated by localized cooling, and thus attenuate finger vasoconstriction. We also anticipated that the cold-evoked regional pain and discomfort would be alleviated after acclimation.

## METHODOLOGY

### Ethics Approval

The experimental protocol was approved by the Human Ethics Committee of Stockholm (2019-05729) and conformed to the standards set by the Declaration of Helsinki. Subjects were informed in detail about the experimental procedures before giving their written consent to participate and were aware that they could terminate their participation at any time.

### Subjects

We recruited 14 healthy men, who, based on their age, anthropometric characteristics, and fitness level, were assigned to either the cold-acclimation [CA, *n* = 7; mean (range) age: 25 (22–28) yr, body mass: 80.2 (66.3–91.3) kg, height: 184 (173–195) cm, body surface area: 2.03 (1.86–2.24) m^2^, total skinfold thickness: 88 (48–124) mm, body fat: 12.2 (6.3–17.5)%, right-hand volume: 428 (353–478) mL, peak oxygen (O_2_) uptake 3.01 (2.35–3.89) L·min^−1^], or the control [CON, *n* = 7; mean (range) age: 26 (23–30) yr, body mass: 75.1 (59.3–86.8) kg, height: 179 (170–187) cm, body surface area: 1.94 (1.69–2.12) m^2^, total skinfold thickness: 83 (54–114) mm, body fat: 11.6 (6.9–16.6)%, right-hand volume: 396 (344–451) mL, and peak O_2_ uptake: 3.10 (2.49–3.66) L·min^−1^] group. No differences were noted between groups in any of these variables (*P* > 0.05). The sample size of the study was based on a previous work that investigated the impact of a cold-acclimation regimen on finger vasomotion (26). All subjects were nonsmokers, normotensive, were not taking any medication, and had no history of cold injury. Subjects resided in Stockholm for ≥18 mo before the initiation of the study, were physically active on a recreational basis, and were not exposed regularly to cold water. Thirteen of them were right handed, and one, who participated in the CA group, was left handed.

### Study Design

The study was performed during winter (December to February; *n*: CA = 4 and CON = 4) and spring (March to June; *n*: CA = 3 and CON = 3) in a laboratory of the Division of Environmental Physiology, Royal Institute of Technology (Solna, Sweden). Approximately a week before the main trials, all subjects attended a preliminary visit, during which they were thoroughly familiarized with the experimental procedures. Anthropometric measures [including body mass, height, percentage body fat (using seven skinfold measurements) (35), body surface area (using the equation by Du Bois and Du Bois) (36), and volume of the right hand (using a water displacement method)] and a graded (25 W·min^−1^) cycling to exhaustion to determine their aerobic capacity were also conducted. Thereafter, subjects performed, on two different consecutive days, a local cold provocation trial consisting of a 30-min hand immersion in 8°C water while immersed to the chest once in 21°C (mild-hypothermic trial) and on the other occasion in 35.5°C (normothermic trial) water (33). Two days after the latter trial, the CA group undertook a 5-day cold-acclimation regimen, during which subjects were immersed to the chest in 14°C water for ≤120 min daily. During the intervention period, the CON group was instructed to avoid any cold exposure. Two and three days after the completion of the cold-acclimation regimen, both groups repeated the two-hand cold provocation trials. In both testing periods, the mild-hypothermic trial was always performed first, because, in the normothermic trial, the immersion period preceding the hand cold provocation was determined by the time required to induce a 0.5°C reduction in rectal temperature (*T*_rec_) in the mild-hypothermic trial (see *Local provocation trials*). For the individual subject, the four trials were performed at the same time of the day [i.e., four subjects from each group conducted the trials in the morning (0830), whereas the rest in the early afternoon (1300)].

During all testing trials and acclimation sessions, subjects were clad in regular swim shorts. Subjects were instructed to *1*) abstain from alcohol and strenuous exercise for at least 24 h before each trial, *2*) refrain from caffeine during the testing day, and *3*) maintain their habitual sleep (≥7 h) and eating routines throughout the experimental period. The environmental conditions in the laboratory were maintained constant throughout the testing trials and acclimation sessions: the mean (standard deviation; SD) temperature, relative humidity, and barometric pressure were 27.1 (0.5)°C, 30 (6)%, and 755 (9) mmHg. The temperature of the water in the immersion tanks was monitored continuously by thermistors (PT100, Texas Instrument, Dallas, TX) placed ∼10–15 cm from the body, at different water depths. If necessary, the water temperature was adjusted by adding ice, or cold tap water.

#### Local cold provocation trials.

Before each trial, subjects were accustomed to the laboratory ambient conditions for ∼30 min, while instrumentation was conducted. Each provocation trial commenced with a 20-min baseline, during which subjects remained in a resting, semireclining position on a gurney placed next to the tank. Thereafter, subjects entered the tank, which was filled with stirred water maintained at 21°C in the mild-hypothermic trials, or at 35.5°C in the normothermic trials. Subjects were immersed to the level of the xiphoid process and remained in a semiupright sitting position with both arms being supported at the level of the heart, above the water surface. The left hand was exposed to the ambient room temperature throughout. The right hand (up to ∼15 cm above the wrist) was placed in a custom-made, water-perfused, tube-lined mitten; warm water was circulated through the tubes maintaining the skin temperature of the fingers at ∼35.5°C (KTH4H/B, Panasonic, Aichi, Japan). During the mild-hypothermic trials, subjects rested idle in this position until their *T*_rec_ dropped by 0.5°C from the baseline value (B-WI phase). In the normothermic trials, since *T*_rec_ remained unchanged, the duration of B-WI phase was similar to that obtained in the preacclimation mild-hypothermic trial. At the end of each B-WI phase, the right hand was removed from the mitten, was covered with a thin plastic bag, and was immersed up to the ulnar and radial styloids for 30 min in a different tank filled with 8°C water (H-CWI phase). After the completion of the H-CWI phase, the right hand was removed from the water, dried with a towel, if necessary, and a 15-min spontaneous hand rewarming period ensued (H-RW phase), during which subjects remained in the tank with both arms resting on the arm-support and being exposed to the room temperature. Then, subjects were removed from the tank, placed in a well-insulated sleeping bag on the gurney, and were monitored for a further 30-min period (B-RW phase). *T*_rec_, skin temperatures, skin blood flux, respiratory gases, arterial pressures, heart rate (HR), and thermoperception were monitored throughout (see Instrumentation).

#### Cold-acclimation sessions.

At the beginning of each session, subjects lay adjacent to the immersion tank in a thermoneutral air (∼27°C) environment for 20 min, while baseline values were recorded. Then, subjects were immersed to the level of the xiphoid process in the tank that was filled with stirred water maintained at 14°C. During the immersion, both arms were supported at the level of the heart, above the water surface; the left hand was exposed to ambient room temperature, whereas the right hand was placed in the mitten maintaining the surface temperature of the fingers at ∼35.5°C, as described previously. The immersion duration was 120 min; but it was terminated earlier if *T*_rec_ dropped below 35°C. On completion of the immersion, subjects were removed directly to a warm shower. *T*_rec_ and skin temperatures, respiratory gases, arterial pressures, HR, and thermoperception were monitored throughout (see *Instrumentation*).

### Instrumentation

#### Thermometry.

*T*_rec_ was monitored continuously with a rectal thermistor (Yellow Springs Instruments, Yellow Springs, OH) placed in a protective sheath and inserted 10 cm beyond the anal sphincter. Mean skin temperature (*T*_sk_) was derived from the unweighted average of skin temperatures, recorded with copper-constantan (T-type) thermocouple (each conductor was 0.2 mm in diameter) probes (Physitemp Instruments Inc., Clifton, NJ) at the left side of the forehead, upper arm, upper and low back, forearm, ring finger, chest, abdomen, thigh, calf, foot, and big toe. Mean body temperature (${\bar{T}}_{\text{b}}$) was calculated by the equation: ${\bar{T}}_{\text{b}}$ = 0.64 × *T*_rec_ + 0.36 × *T*_sk_ (37). Five additional thermocouples were attached to the middle of the palmar side of the distal phalanx of each finger of the right hand. The primary insulation of the thermocouples was polytetrafluoroethylene; the noninsulated welded junctions of the thermocouple were attached directly to the skin with thin air-permeable tape. All temperatures were sampled at 1 Hz with a NI USB-6215 data acquisition system, and processed with LabVIEW software (v. 2019, National Instruments, Austin, TX). Before each trial, all temperature probes were calibrated against a certified reference thermometer (Ellab, Copenhagen, Denmark).

The average (*T*_F-avg_), minimum, and maximum temperature of each finger of the right hand obtained during each phase was calculated from the thermocouple measures using a custom-made computer program based on TestPoint (v7, Norton, MA). The same program was also used to detect any finger CIVD event, defined as a local skin-temperature wave in terms of ≥1°C increase lasting for a minimum duration of 3 min. In case of a CIVD event, the following parameters were determined: *1*) the temperature amplitude, which was the difference between the lowest temperature recorded just before the CIVD and the highest temperature reached during the CIVD and *2*) the duration of a local skin temperature wave.

#### Respiratory measurements.

Subjects were equipped with a facemask to enable the breath-by-breath monitoring of O_2_ uptake, CO_2_ production, and respiratory exchange ratio, by means of a metabolic unit (Quark PFT; Cosmed, Rome, Italy). The gas analyzers and pneumotachograph were calibrated before each session with two different gas mixtures (room air: 20.93% O_2_ and 0.04 CO_2_, and certified gas mixture: 16.00% O_2_ and 5.00% CO_2_) and a 3-L syringe, respectively. The individual shivering thresholds, indicated by a sustained elevation in O_2_ uptake [i.e., visual identification of >100 mL·min^−1^ increase from the initial plateau observed upon immersion (38)], were derived from the responses of O_2_ uptake relative to changes in *T*_rec_. Metabolic heat production ($\dot{M}$) was calculated by the equation: $\dot{M}$ = (0.23 × respiratory exchange ratio + 0.77) × 5.873 O_2_ uptake × 60, and reported in Watts.

#### Arterial pressures and heart rate.

Beat-to-beat systolic (SAP), diastolic (DAP), and mean (MAP) arterial pressures were measured continuously using a volume-clamp technique (Finometer, Finapres Medical Systems BV, Amsterdam, The Netherlands), with the pressure cuff placed around the middle phalanx of the left middle finger, and with the reference pressure transducer positioned at the level of the heart. The Finometer-derived values were verified intermittently by electro-sphygmomanometry (Omron, M6, Kyoto, Japan). HR was derived from the arterial pressure curves as the inverse of the interbeat interval.

#### Skin blood flux.

Local skin blood flux was monitored continuously at a rate of 10 Hz on the palmar side of the distal phalanx of the right index finger by laser-Doppler flowmetry (VMS-LDF2; Moor Instruments, Axminster, UK) using optic probes (VP1/7; Moor Instruments, UK), which was firmly connected to the skin with double-sided adhesive tape. Skin blood flux was reported as cutaneous vascular conductance (CVC), calculated as skin blood flux divided by MAP.

#### Perceptual measurements.

During the baseline, B-WI (at 10-min intervals), H-CWI (at *minutes 1*, *2*, *3*, *4*, *5,* and every 5 min thereafter), H-RW (at *minutes 1*, *5*, *10*, and *15*), and B-RW (at 10-min intervals) phases in the provocation trials, subjects were asked to provide ratings of their whole body and right-hand thermal sensation (from 1-cold to 7-hot) and thermal comfort (from 1-comfortable to 4-very uncomfortable). At the same time intervals, the general affective valence (from −5-very bad to +5-very good), the perceived shivering intensity (from 1-no shivering to 4-heavy shivering), and the local (right hand) pain (from 0-no pain to 10-unbearable pain) were also assessed. During the acclimation sessions, the same perceptual measurements were performed at 10-min intervals.

### Statistical Analyses

Baseline values were calculated as averages of the final 10 min of the 20-min baseline phase. All physiological data were reduced to 60 s averages. For all datasets, normality of distribution was assessed using the Shapiro–Wilk test. To evaluate changes between the acclimation sessions, a one-way repeated-measures analysis of variance (ANOVA) was used for all physiological variables. Mauchly’s test was conducted to assess the sphericity and, if necessary, the Greenhouse–Geiser ε correction was used to adjust the degrees of freedom. When ANOVA revealed significant effects, changes from the 1st acclimation session were assessed with Dunnett’s post hoc test. Differences in the perceptual responses between the acclimation sessions were evaluated with Friedman’s test, followed by the Wilcoxon signed-rank test. To evaluate differences between the provocation trials, a two-way [group (CA × CON) × testing period (pre × post)] ANOVA was used for all physiological variables. When an ANOVA revealed a significant *F* value, multiple pairwise comparisons were performed with Duncan’s multiple range test. The perceptual responses during the provocation trials were compared with Kruskal–Wallis test, followed by either the Mann–Whitney *U* (between-groups comparison) or the Wilcoxon signed-rank (within-group comparison) tests. Effect sizes are reported as partial eta-squared (${\text{η}}_{\text{p}}^{\text{2}}$; values of ≤0.02, ≤0.13, and ≥0.26 are considered as small, moderate, and large, respectively). Furthermore, effect sizes were calculated using Cohen’s *d* (values of ≤0.2, ≤0.5, and ≥0.8 are considered as small, moderate, and large, respectively) and *r* (values of ≤0.1, ≤0.3, and ≥0.5 are considered as small, moderate, and large, respectively) for the parametric and nonparametric pairwise comparisons, respectively. Statistical analyses were conducted using Statistica 8.0 (StatSoft, Tulsa, OK), and figures were produced using Prism 9.2 (GraphPad Software, Inc., San Diego, CA). Unless otherwise stated, data are presented as mean values with 95% confidence intervals [CI], which were calculated using a noncentral *t* distribution. The α level of significance was set a priori at 0.05.

## RESULTS

### Cold-Acclimation Regimen

**Figure 1.**
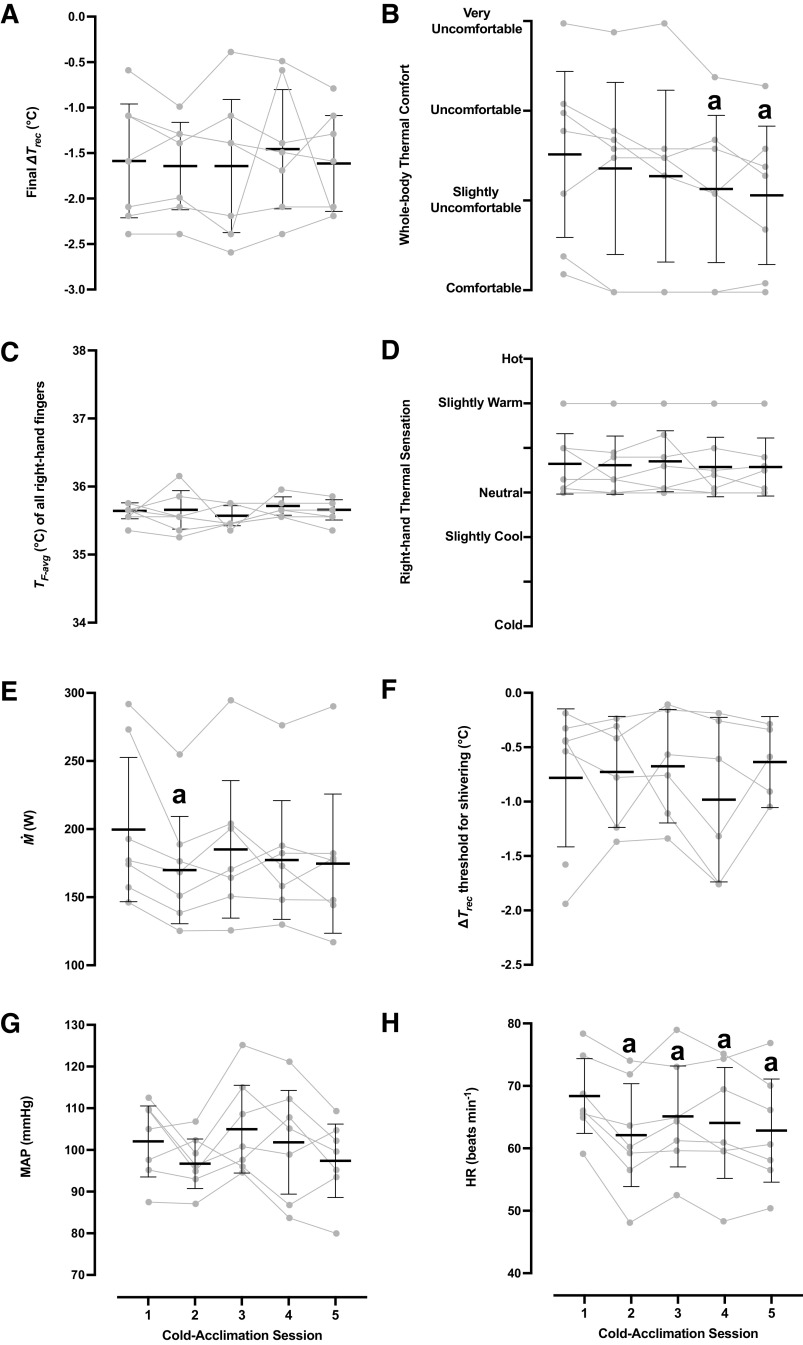
Physiological and perceptual responses obtained during the 5-day cold-acclimation regimen (*n* = 7 men). Mean [95% confidence interval] and individual values of the final changes in rectal temperature (Δ*T*_rec_; changes are relative to baseline values) (*A*), whole body thermal comfort (*B*), skin temperature of the right-hand fingers (*T*_F-avg_) (*C*), right-hand thermal sensation (*D*), metabolic heat production ($\dot{M}$) (*E*), shivering thresholds (*F*), mean arterial pressure (MAP) (*G*), and heart rate (HR) (*H*). Physiological data were analyzed with one-way repeated-measures ANOVA, followed by Dunnett’s post hoc test. Perceptual data were analyzed with Friedman’s test, followed by Wilcoxon signed-rank test (*P* ≤ 0.05). ^a^Significantly different from the 1st session.

The physiological and perceptual responses obtained during each acclimation session are summarized in Table 1 and Fig. 1. All seven subjects completed the 5-day acclimation regimen. Three subjects terminated the 120-min immersion prematurely (one subject in all five sessions, one in the last four sessions, and one in the 5th session), because their *T*_rec_ reached the critical temperature of 35°C. Neither the mean duration of the immersion (*P* = 0.50; ${\text{η}}_{\text{p}}^{\text{2}}$ = 0.12), nor the cooling rate (*P* = 0.61, ${\text{η}}_{\text{p}}^{\text{2}}$ = 0.10) differed between sessions. Overall, *T*_rec_ dropped by ∼1.5°C daily, whereas *T*_F-avg_ was maintained at ∼35.6°C throughout. During the 14°C immersions, subjects consistently felt their right hand slightly warm, thermally comfortable, and pain-free.

**Table 1.** Physiological and perceptual responses obtained during the 5-day cold-acclimation regimen (n = 7 men)

	Cold-Acclimation Session				
	1st	2nd	3rd	4th	5th
Water temperature, °C	14.1 (14.0–14.3)	14.1 (14.0–14.2)	14.1 (14.0–14.3)	14.0 (14.0–14.1)	14.0 (14.0–14.1)
Duration of immersion, min	111 (60–120)	107 (63–120)	110 (75–120)	103 (59–120)	97 (36−120)
Cooling rate, °C·h^-1^	–0.48 [–0.18, –0.78]	–0.49 [–0.18, –0.79]	−0.45 [−0.14, −0.76]	−0.54 [−0.12, −0.95]	−0.62 [−0.14, −1.10]
Baseline *T*_rec_, °C	37.4 [37.2, 37.6]	37.3 [37.1, 37.5]	37.4 [37.2, 37.6]	37.3 [37.1, 37.4]	37.2 [37.0, 37.4]
Final *T*_rec_, °C	35.8 [35.2, 36.4]	35.7 [35.2, 36.2]	35.7 [35.1, 36.4]	35.8 [35.2, 36.5]	35.6 [34.9, 36.2]
Baseline *T*_sk_, °C	33.5 [33.1, 34.0]	33.3 [32.8, 33.8]	33.4 [32.9, 33.8]	33.3 [32.8, 33.8]	33.1 [32.4, 33.8]
Final *T*_sk_, °C	22.3 [21.9, 22.6]	22.2 [21.8, 22.7]	21.9 [21.5, 22.3]	22.1 [21.7, 22.5]	22.2 [21.4, 23.0]
Final ${\bar{T}}_{\text{b}}$, °C	30.9 [30.5, 31.4]	30.8 [30.5, 31.1]	30.8 [30.4, 31.1]	30.9 [30.4, 31.4]	30.8 [30.2, 31.3]
SAP, mmHg	134 [123, 145]	128 [119, 137]	138 [124, 152]	135 [121, 149]	129 [118, 140]^a^
DAP, mmHg	80 [73, 88]	75 [69, 81]	82 [74, 90]	79 [69, 89]	76 [69, 83]
Perceived shivering	2.2 (1–3)	1.9 (1–3)^a^	1.9 (1–3)	1.8 (1–3)^a^	1.7 (1–2)
Body thermal sensation	1.9 (1–3)	2.1 (1–4)	2.1 (1–4)	2.2 (1–4)^a^	2.2 (1–4)^a^
Body thermal pain	2.4 (0–5)	1.7 (0–4)^a^	2.1 (0–6)	1.7 (0–5)	1.3 (0–4)^a^
Right-hand thermal comfort	1	1	1	1	1
Affective valence	0.3 (−3 to 4)	0.6 (−4 to 4)	0.6 (−4 to 4)	1.0 (−3 to 5)^a^	1.2 (−2 to 5)^a^

Values are represented as means [95% confidence interval] for cooling rate, rectal (*T*_rec_), skin (*T*_sk_), and mean body (${\bar{T}}_{\text{b}}$) temperatures, and systolic (SAP) and diastolic (DAP) arterial pressures. Values are mean (range) for water temperature, duration of immersion, perceived shivering, thermal sensation, pain and comfort, and affective valence. Physiological data were analyzed with one-way repeated-measures ANOVA, followed by Dunnett’s post hoc test. Perceptual data were analyzed with Friedman’s test, followed by Wilcoxon signed-rank test (*P* ≤ 0.05).

^a^ Significantly different from the 1st session.

The cold-induced elevation (*P* < 0.001) in $\dot{M}$ was gradually attenuated across the acclimation regimen, especially during the 2nd session (*P* = 0.03). HR was persistently lower in the last four sessions than in the 1st session (*P* < 0.001). The perceived shivering intensity was also slightly diminished during the 2nd and 4th sessions (*P* = 0.05). Subjects felt less unpleasant (*P* = 0.001), and the whole body sensation of coldness and thermal discomfort were blunted (*P* ≤ 0.05) in the final two sessions.

### Local Cold Provocation

#### Mild-hypothermic trial.

*T*_rec_, *T*_sk_, and ${\bar{T}}_{\text{b}}$, which did not vary between the baseline phases, dropped in all trials (*P* < 0.001), and to a similar extent (*P* > 0.05; Table 2). The duration of the B-WI phase (i.e., when *T*_rec_ dropped 0.5°C) did not differ between trials [mean (range) for the CA group: Pre = 82 (25–150) min, Post = 69 (24–125) min, and for the CON group: Pre = 66 (49–87) min, Post = 63 (36–107) min; *P* = 0.45, ${\text{η}}_{\text{p}}^{\text{2}}$ = 0.04].

**Table 2.** Physiological and perceptual responses obtained during the baseline, the body water-immersion, the 30-min hand cold-water immersion, the 15-min hand-rewarming, and the 30-min body-rewarming phases in the mild-hypothermic provocation trials, performed before and after the 5-day acclimation regimen

	Preacclimation					Postacclimation				
	Baseline	B-WI	H-CWI	H-RW	B-RW	Baseline	B-WI	H-CWI	H-RW	B-RW
CA group										
*T*_rec_, °C	37.3 [37.1, 37.4]	37.1 [36.9, 37.2]	36.6 [36.4, 36.8]	36.4 [36.0, 36.7]	36.2 [35.7, 36.8]	37.1 [36.8, 37.4]	36.9 [36.7, 37.2]	36.5 [36.2, 36.7]	36.3 [36.0, 36.6]	36.1 [35.6, 36.6]
*T*_sk_, °C	33.1 [32.6, 33.6]	26.7 [26.6, 26.8]	26.2 [25.9, 26.5]	26.1 [25.8, 26.4]	28.3 [27.9, 28.7]	33.6 [33.3, 33.8]	26.7 [26.3, 27.1]	26.1 [25.7, 26.5]	26.1 [25.8, 26.4]	28.1 [27.7, 28.6]
${\bar{T}}_{\text{b}}$, °C	35.8 [35.6, 35.9]	33.3 [33.3, 33.4]	32.8 [32.7, 33.0]	32.7 [32.4, 32.9]	33.4 [33.0, 33.7]	35.8 [35.6, 36.1]	33.3 [33.1, 33.4]	32.8 [32.6, 32.9]	32.6 [32.4, 32.8]	33.2 [32.9, 33.6]
$\dot{M}$, W	129 [110, 148]	142 [120, 164]	187 [149, 225]	176 [136, 216]	136 [122, 150]	128 [112, 143]	136 [121, 150]	163 [129, 197]*	168 [126, 210]	134 [117, 152]
SAP, mmHg	127 [113, 142]	130 [123, 137]	141 [133, 150]	140 [130, 149]	138 [129, 147]	127 [120, 134]	131 [123, 140]	133 [126, 140]	128 [117, 138]	142 [133, 151]
DAP, mmHg	73 [62, 85]	73 [68, 77]	85 [78, 92]	85 [78, 91]	83 [74, 92]	77 [69, 84]	77 [72, 82]	81 [78, 84]	80 [75, 84]	88 [84, 91]
MAP, mmHg	92 [80, 105]	95 [89, 100]	109 [100, 118]	109 [101, 117]	106 [96, 115]	95 [87, 102]	98 [92, 104]	103 [98, 109]	101 [94, 108]	110 [105, 116]
HR, beats/min	70 [66, 75]	62 [57, 68]	63 [56, 71]	60 [52, 68]	57 [51, 63]	71 [59, 83]	63 [53, 72]	62 [53, 70]	60 [52, 68]	57 [48, 66]
Perceived shivering	1	1.6 (1–3)	2.4 (1–4)	2.0 (1–3)	1.2 (1–2)	1	1.2 (1–2)	1.9 (1–3)	1.9 (1–3)	1.2 (1–2)
Body thermal sensation	5.3 (4–6)	2.5 (1–3)	2.7 (2–4)	2.8 (2–4)†	3.9 (3–5)	5.1 (4–6)	3.2 (2–4)*†	3.4 (3–4)	3.0 (2–4)†	4.3 (4–6)*†
Body thermal comfort	1	1.8 (1–3)	2.0 (1–3)	1.6 (1–3)	1.1 (1–2)	1	1.4 (1–2)*†	1.3 (1–2)	1.6 (1–3)†	1.2 (1–2)
Affective valence	2.3 (0 to 5)	1.2 (−2 to 5)	−0.4 (−3 to 3)	0.6 (−1 to 3)	2.5 (−1 to 4)	2.6 (0 to 5)	2.1 (0 to 5)*	0.9 (−2 to 5)*	1.0 (−3 to 5)	1.6 (−2 to 5)
CON group										
*T*_rec_, °C	37.2 [37.0, 37.3]	37.0 [36.9, 37.1]	36.4 [36.1, 36.6]	36.0 [35.6, 36.5]	35.8 [35.2, 36.4]	37.2 [37.0, 37.3]	36.9 [36.7, 37.1]	36.4 [36.3, 36.6]	36.2 [36.0, 36.4]	35.9 [35.6, 36.2]
*T*_sk_, °C	33.1 [32.4, 33.8]	26.9 [26.5, 27.3]	26.3 [25.9, 26.7]	26.1 [25.7, 26.5]	28.3 [27.7, 28.8]	33.4 [33.1, 33.7]	26.7 [26.3, 27.1]	26.2 [25.8, 26.7]	26.1 [25.7, 26.5]	28.3 [27.6, 29.0]
${\bar{T}}_{\text{b}}$, °C	35.7 [35.4, 36.0]	33.4 [33.2, 33.5]	32.7 [32.5, 33.0]	32.5 [32.2, 32.8]	33.1 [32.7, 33.5]	35.8 [35.6, 36.0]	33.3 [33.0, 33.5]	32.8 [32.5, 33.0]	32.6 [32.3, 32.8]	33.2 [32.9, 33.5]
$\dot{M}$, W	125 [115, 136]	133 [119, 147]	180 [131, 229]	202 [157, 247]	153 [134, 172]	123 [109, 138]	132 [114, 150]	147 [115, 179]*	159 [123, 194]*	134 [120, 148]*
SAP, mmHg	125 [113, 137]	130 [116, 144]	144 [121, 168]	139 [119, 159]	139 [125, 153]	119 [107, 131]	124 [116, 132]	135 [123, 148]	135 [119, 151]	138 [130, 147]
DAP, mmHg	73 [68, 79]	71 [62, 79]	82 [69, 96]	81 [71, 90]	83 [77, 88]	70 [64, 77]	68 [66, 70]	78 [73, 82]	78 [72, 83]	85 [82, 88]
MAP, mmHg	92 [85, 100]	93 [84, 103]	109 [92, 126]	106 [94, 118]	106 [99, 114]	88 [80, 96]	89 [86, 93]	102 [96, 107]	102 [95, 108]	108 [105, 110]
HR, beats/min	69 [62, 75]	60 [56, 64]	64 [54, 74]	63 [56, 71]	56 [51, 60]	70 [64, 77]	59 [56, 64]	60 [52, 67]	58 [51, 66]	54 [47, 60]
Perceived shivering	1	1.3 (1–2)	2.1 (1–3)	2.2 (1–4)	1.4 (1–2)	1	1.1 (1–2)	1.6 (1–3)*	1.9 (1–3)	1.1 (1–2)
Body thermal sensation	5.0 (4–6)	2.2 (2–3)	2.1 (1–3)	1.9 (1–3)	3.2 (2–4)	4.6 (4–6)	2.4 (2–3)	2.5 (1–4)	2.0 (1–3)	3.3 (2–5)
Body thermal comfort	1	2.0 (2)	2.2 (1–3)	2.4 (2–3)	1.3 (1–2)	1	1.9 (1–2)	2.0 (1–3)	2.2 (2–3)	1.3 (1–2)
Affective valence	3.3 (0 to 5)	2.1 (0 to 4)	0.1 (−1 to 4)	0.4 (−2 to 4)	2.0 (0 to 5)	2.9 (0 to 5)	2.3 (0 to 5)	0.9 (−2 to 5)	1.0 (−1 to 4)	2.1 (0 to 5)

Data are the mean of the entire phase. Values are mean [95% confidence intervals] for rectal (*T*_rec_), skin (*T*_sk_), and mean body (${\bar{T}}_{\text{b}}$) temperatures, metabolic heat production ($\dot{M}$), systolic (SAP), diastolic (DAP) and mean (MAP) arterial pressures, and heart rate (HR). Values are mean (range) for perceived shivering, thermal sensation and comfort, and affective valence. Physiological data were analyzed with two-way repeated-measures ANOVA, followed by Duncan’s multiple range test. Perceptual data were analyzed with Kruskal–Wallis’ test, followed by either Mann–Whitney *U* or Wilcoxon signed-rank test (*P* ≤ 0.05). B-WI, body water-immersion; B-RW, 30-min body-rewarming; CA group, cold-acclimation group (*n* = 7 men); CON group, control group (*n* = 7 men). H-CWI, 30-min hand cold-water immersion; H-RW, 15-min hand-rewarming.

Significantly different from the preacclimation trial (*), and from the CON group(†).

**Figure 2.**
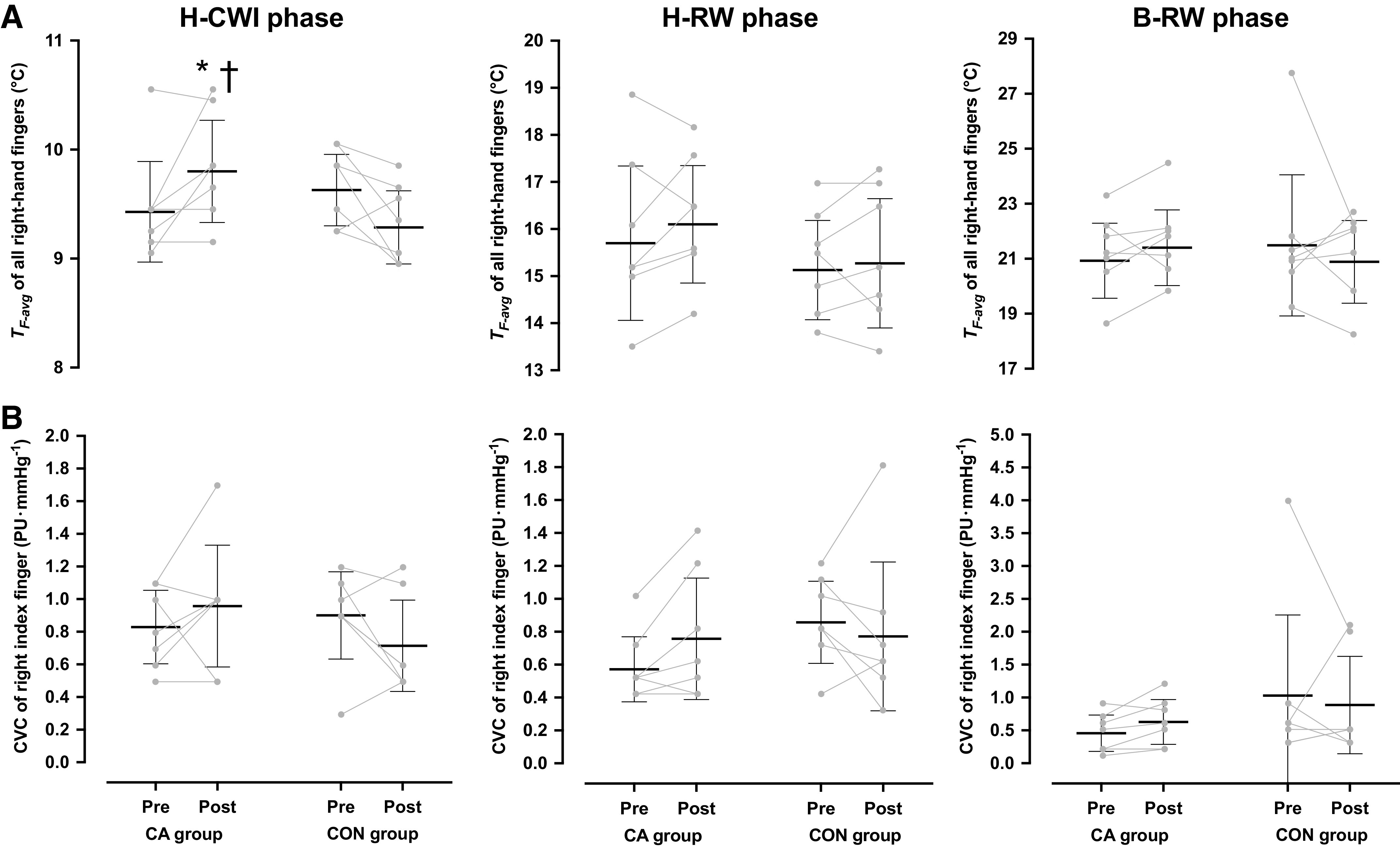
Mean [95% confidence interval] and individual skin temperature (*T*_F-avg_) of all fingers (*A*) and cutaneous vascular conductance (CVC) of the index finger (*B*) of the right hand obtained during the 30-min hand cold-water immersion (H-CWI), the 15-min hand-rewarming (H-RW), and the 30-min body-rewarming (B-RW) phases in the mild-hypothermic provocation trial, performed before and after the 5-day acclimation regimen. CA group, cold-acclimation group (*n* = 7 men); CON group, control group (*n* = 7 men). Data were analyzed with two-way repeated-measures ANOVA, followed by Duncan’s multiple range test (*P* ≤ 0.05). Significantly different from the preacclimation trial (*) and from the CON group (†).

During all B-WI phases, *T*_F-avg_ was maintained at ∼35.5°C (CA group: Pre = 35.6 [35.6, 35.7]°C, Post = 35.7 [35.7, 35.8]°C, and CON group: Pre = 35.6 [35.3, 35.8]°C, Post = 35.7 [35.5, 35.8]°C; *P* = 0.98, ${\text{η}}_{\text{p}}^{\text{2}}$ < 0.001). *T*_F-avg_ dropped during all H-CWI phases (*P* < 0.001); yet the reduction in *T*_F-avg_ was less in the post-CA H-CWI than in the pre-CA (*P* = 0.05, *d* = 1.90) and post-CON (*P* = 0.05, *d* = 0.88) H-CWIs (Fig. 2*A*). The regimen did not modify the incidence of CIVD events in the CA group (*P* = 0.31, *d* = 1.57), whereas the CON group displayed no CIVD events, either in the pre- or in the posttrials (Table 3). The minimum finger temperature was higher in the post-CA than in the pre-CA (*P* = 0.02) and post-CON (*P* = 0.03) H-CWIs (Table 3). The maximum finger temperature, as well as the temperature amplitude and the duration of the CIVD events did not differ between trials (*P* > 0.05; Table 3). No intertrial differences were noted in *T*_F-avg_ across the rewarming phases (H-RW: *P* = 0.61, ${\text{η}}_{\text{p}}^{\text{2}}$ = 0.02 and B-RW: *P* = 0.32, ${\text{η}}_{\text{p}}^{\text{2}}$ = 0.08; Fig. 2*A*).

**Table 3.** Minimum and maximum finger temperature, number of cold-induced vasodilatation events, temperature amplitude, and duration of cold-induced vasodilatation on the palmar side of the distal phalanx of the righthand fingers during the 30-min hand cold-water immersion phase in the mild-hypothermic and the normothermic provocation trials, performed before and after the 5-day acclimation regimen

	CA Group			CON Group	
	Preacclimation	Postacclimation		Preacclimation	Postacclimation
Mild-hypothermic trial					
Minimum temperature, °C	8.3 [7.8, 8.7]	8.6 [8.2, 9.1]*†		8.2 [8.0, 8.5]	8.1 [7.9, 8.3]
Maximum temperature, °C	13.9 [12.5, 15.3]	15.1 [12.5, 17.7]		15.5 [13.8, 17.1]	14.8 [12.9, 16.8]
CIVD events (no.)	1 (7)	3 (21)		0	0
CIVD amplitude, °C	0.2 [−0.2, 0.6]	1.3 [−0.8, 3.5]		-	-
CIVD duration, min	1.5 [−1.1, 4.2]	3.0 [0.5, 5.4]		-	-
Normothermic trial					
Minimum temperature, °C	8.8 [8.3, 9.3]	9.3 [8.8, 9.8]*†		8.8 [8.2, 9.4]	8.5 [7.9, 9.1]
Maximum temperature, °C	16.9 [15.9, 17.9]	16.7 [13.8, 19.5]		15.6 [13.5, 17.7]	15.0 [13.4, 16.7]
CIVD events (no.)	6 (42)	8 (59)†		4 (27)	2 (17)
CIVD amplitude, °C	2.2 [0.5, 3.8]	2.9 [0.8, 5.1]		1.0 [0.1, 2.0]	1.0 [0.0, 2.0]
CIVD duration, min	5.4 [2.1, 8.7]	5.9 [−0.2, 6.9]		3.3 [2.9, 9.0]	2.7 [0.1, 5.3]

Values are mean [95% confidence interval]. Values for CIVD are mean (total incidence). Data were analyzed with two-way repeated-measures ANOVA, followed by Duncan’s multiple range test (*P* ≤ 0.04). CA group: cold-acclimation group (*n* = 7 men); CIVD, cold-induced vasodilatation; CON group: control group (*n* = 7 men).

Significantly different from the preacclimation trial (*) and from the CON group (†).

The right-finger CVC was similar in all trials, both in the baseline (CA group: Pre = 2.9 [1.4, 4.4] PU·mmHg^−1^, Post = 3.3 [2.6, 4.0] PU·mmHg^−1^, and CON group: Pre = 3.1 [2.5, 3.6] PU·mmHg^−1^, Post = 3.5 [3.0, 4.0] PU·mmHg^−1^; *P* = 0.92, ${\text{η}}_{\text{p}}^{\text{2}}$ < 0.001) and the B-WI (CA group: Pre = 1.2 [0.2, 2.2] PU·mmHg^−1^, Post = 1.2 [0.8, 1.5] PU·mmHg^−1^, and CON group: Pre = 1.4 [0.8, 1.9] PU·mmHg^−1^, Post = 1.6 [0.9, 2.3] PU·mmHg^−1^; *P* = 0.39, ${\text{η}}_{\text{p}}^{\text{2}}$ = 0.06) phases. The cold-induced reduction in the right-finger CVC did not differ between trials (*P* = 0.13, ${\text{η}}_{\text{p}}^{\text{2}}$ = 0.17; Fig. 2*B*). No intertrial differences were observed in the right-finger CVC, either in the H-RW (*P* = 0.14, ${\text{η}}_{\text{p}}^{\text{2}}$ = 0.16) or in the B-RW (*P* = 0.40, ${\text{η}}_{\text{p}}^{\text{2}}$ = 0.04) phases (Fig. 2*B*).

**Figure 3.**
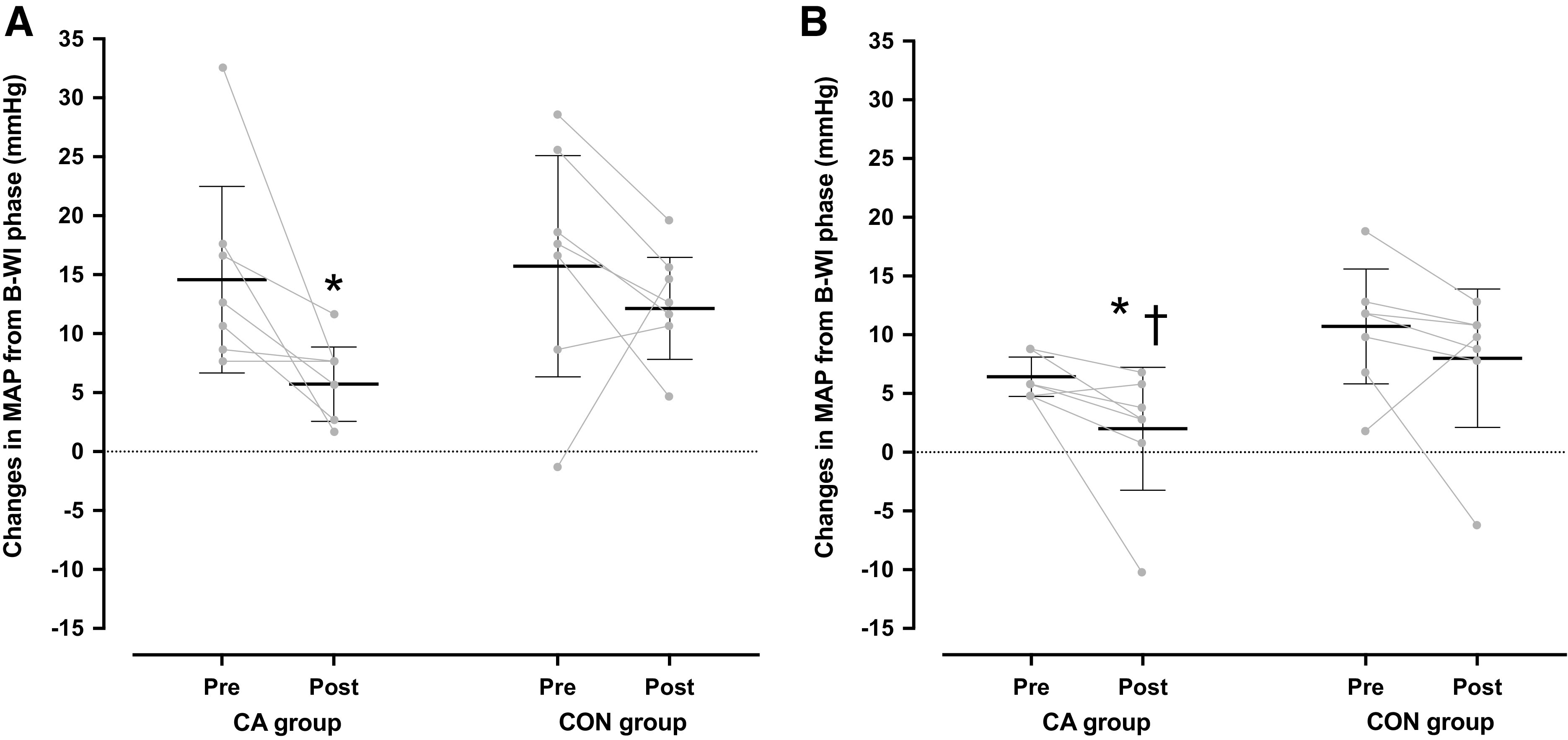
Mean [95% confidence interval] and individual changes in mean arterial pressure during the 30-min hand cold-water immersion (H-CWI) phase in the mild-hypothermic (*A*) and the normothermic provocation (*B*) trials, performed before and after the 5-day acclimation regimen. Changes are relative to the values obtained during the body water-immersion phase. CA group, cold-acclimation group (*n* = 7 men); CON group, control group (*n* = 7 men). Data were analyzed with two-way repeated-measures ANOVA, followed by Duncan’s multiple range test (*P* ≤ 0.05). Significantly different from the preacclimation trial (*) and from the CON group (†).

The arterial pressures and HR did not differ between trials (*P* > 0.05; Table 2). However, during the post-H-CWI phase, the magnitude of MAP elevation elicited by the localized cooling was blunted in the CA group (*P* = 0.03, *d* = 0.58), but not in the CON group (*P* = 0.39, *d* = 0.34) (Fig. 3*A*). In both groups, the cold-induced increase in $\dot{M}$ was attenuated during the posttrials (*P* < 0.05, Table 2).

**Figure 4.**
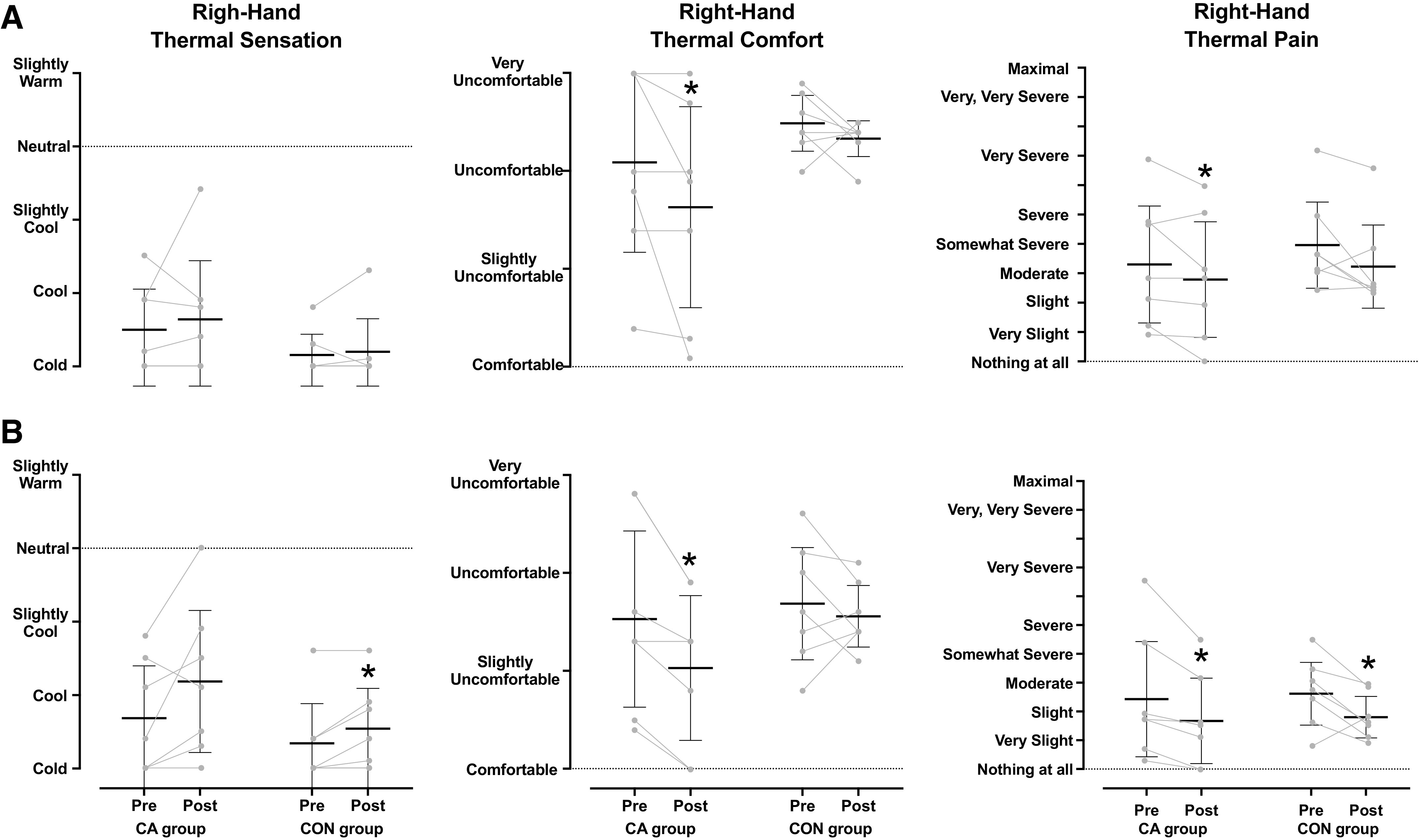
Mean [95% confidence interval] and individual values of right-hand thermal sensation, comfort and pain obtained during the 30-min hand cold-water immersion phase in the mild-hypothermic (*A*) and the normothermic (*B*) provocation trials, performed before and after the 5-day acclimation regimen. CA group, cold-acclimation group (*n* = 7 men); CON group, control group (*n* = 7 men). Data were analyzed with Kruskal–Wallis’ test, followed by either Mann–Whitney *U* or Wilcoxon signed-rank test (*P* ≤ 0.05). *Significantly different from the preacclimation trial.

The regional (right hand) thermoperceptual responses obtained during the H-CWI phase are depicted in Fig. 4*A*. In the posttrials, the right-hand thermal discomfort was attenuated in the CA group (*P* = 0.04, *r* = 2.64) but not in the CON group (*P* = 0.26, *r* = 0.42). After the acclimation, the cold-induced pain was alleviated in the CA group (CA group: *P* = 0.05, *r* = 0.69 and CON group: *P* = 0.06, *r* = 0.69), whereas the regional thermal sensation remained unaltered in both groups (*P* > 0.05). The whole body sensation of coldness and thermal discomfort were attenuated (*P* ≤ 0.04; Table 2), and the rates of affective valence were slightly enhanced (*P* ≤ 0.05) only in the CA group (Table 2).

#### Normothermic trial.

**Figure 5.**
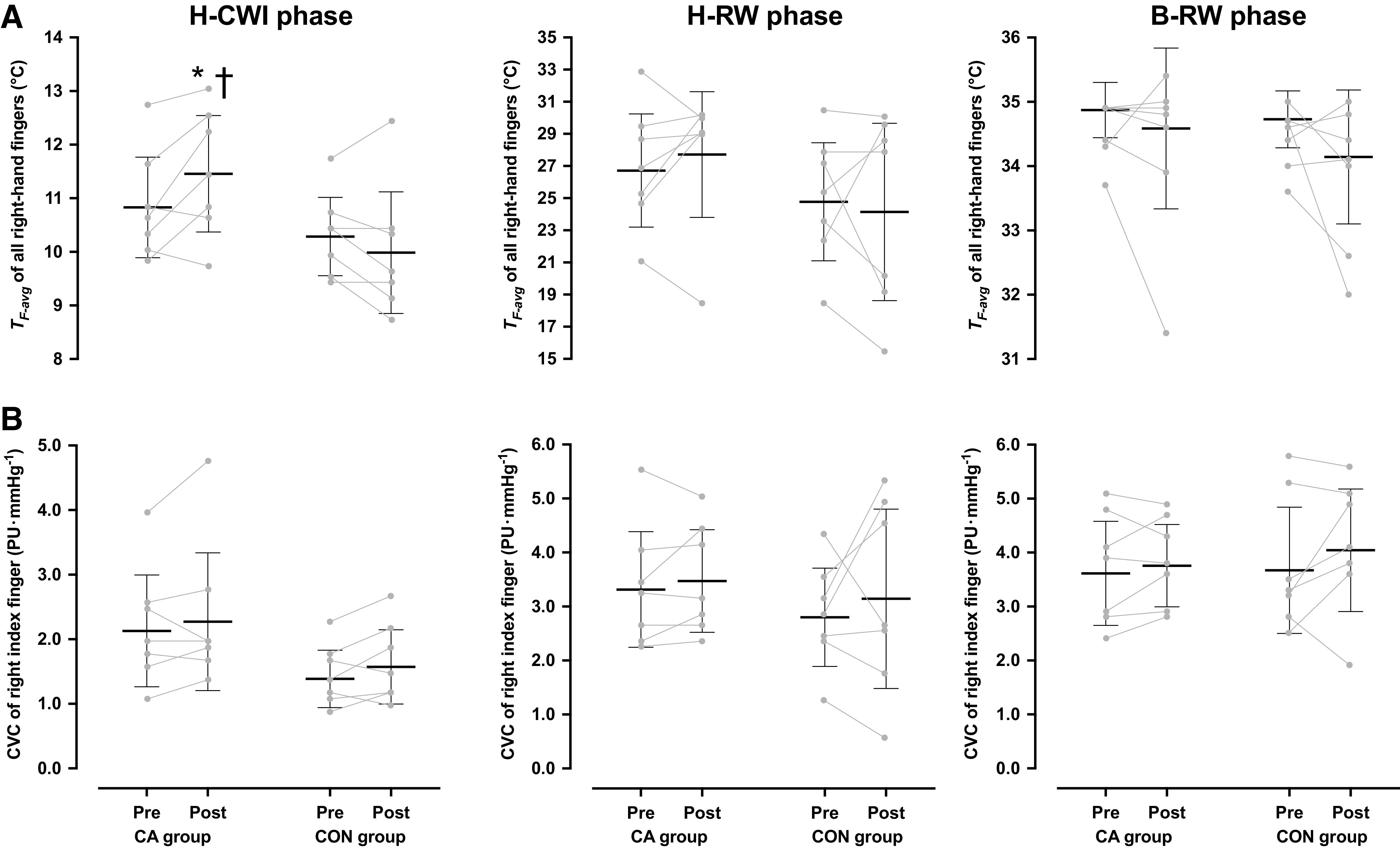
Mean [95% confidence interval] and individual skin temperature (*T*_F-avg_) of all fingers (*A*) and cutaneous vascular conductance (CVC) of the index finger (*B*) of the right hand obtained during the 30-min hand cold-water immersion (H-CWI), the 15-min hand-rewarming (H-RW), and the 30-min body-rewarming (B-RW) phases in the normothermic provocation trial, performed before, and after the 5-day acclimation regimen. CA group, cold-acclimation group (*n* = 7 men); CON group, control group (*n* = 7 men). Data were analyzed with two-way repeated-measures ANOVA, followed by Duncan’s multiple range test (*P* ≤ 0.05). Significantly different from the preacclimation trial (*) and from the CON group (†).

*T*_rec_, *T*_sk_, and ${\bar{T}}_{\text{b}}$ did not differ between trials (*P* > 0.05; Table 4). During the B-WI phases, *T*_F-avg_ was similar in all trials (CA group: Pre = 36.0 [35.9, 36.1]°C, Post = 36.0 [35.8, 36.2]°C, and CON group: Pre = 36.0 [35.9, 36.1]°C, Post = 35.9 [35.8, 36.1]°C; *P* = 0.87, ${\text{η}}_{\text{p}}^{\text{2}}$ < 0.001). *T*_F-avg_ dropped during all H-CWI phases (*P* < 0.001), yet the reduction in *T*_F-avg_ was less in the post-CA than in the pre-CA (*P* = 0.02, *d* = 0.81) and post-CON (*P* = 0.03, *d* = 0.83) H-CWIs (Fig. 5*A*). After the acclimation regimen, the incidence of CIVD events was greater in the CA than in the CON group (*P* = 0.04, *d* = 0.70; Table 3). The minimum finger temperature was higher in the post-CA than in the pre-CA (*P* = 0.02) and post-CON (*P* = 0.02) H-CWIs (Table 3). The maximum finger temperature, as well as the temperature amplitude and the duration of the CIVD events did not differ between trials (*P* > 0.05; Table 3). No intertrial differences were noted in *T*_F-avg_ across the rewarming phases (H-RW: *P* = 0.47, ${\text{η}}_{\text{p}}^{\text{2}}$ = 0.04 and B-RW: *P* = 0.61, ${\text{η}}_{\text{p}}^{\text{2}}$ = 0.02; Fig. 5*A*).

**Table 4.** Physiological and perceptual responses obtained during the baseline, the body immersion, the 30-min hand cold-water immersion, the 15-min hand-rewarming, and the 30-min body-rewarming phases in the normothermic provocation trials, performed before and after the 5-day acclimation regimen

	Preacclimation					Postacclimation				
	Baseline	B-WI	H-CWI	H-RW	B-RW	Baseline	B-WI	H-CWI	H-RW	B-RW
CA group										
* T*_rec_, °C	37.1 [36.9, 37.4]	37.1 [36.9, 37.3]	37.1 [36.9, 37.4]	37.1 [36.8, 37.4]	37.1 [36.9, 37.3]	37.1 [36.9, 37.4]	37.1 [36.9, 37.3]	37.1 [36.9, 37.4]	37.0 [36.8, 37.3]	37.1 [36.8, 37.3]
* T*_sk_, °C	33.9 [33.3, 34.5]	34.9 [34.6, 35.1]	34.8 [34.5, 35.0]	34.8 [34.7, 34.9]	34.6 [34.4, 34.8]	33.8 [33.2, 34.4]	34.8 [34.6, 35.0]	34.6 [34.4, 34.9]	34.6 [34.3, 34.9]	34.6 [34.2, 35.0]
${\bar{T}}_{\text{b}}$, °C	36.0 [35.6, 36.3]	36.3 [36.1, 36.5]	36.3 [36.1, 36.5]	36.3 [36.1, 36.5]	36.2 [36.1, 36.4]	35.9 [35.6, 36.3]	36.3 [36.1, 36.5]	36.2 [36.0, 36.5]	36.2 [36.0, 36.4]	36.2 [35.9, 36.4]
$\dot{M}$, W	128 [107, 148]	132 [107, 157]	128 [106, 149]	124 [104, 145]	127 [110, 144]	126 [113, 139]	129 [116, 143]	128 [117, 138]	126 [114, 137]	122 [108, 137]
SAP, mmHg	123 [116, 131]	108 [101, 114]	117 [110, 124]	114 [107, 121]	121 [111, 130]	121 [111, 130]	105 [92, 118]	110 [100, 121]	110 [102, 119]	121 [108, 135]
DAP, mmHg	74 [67, 80]	62 [56, 69]	67 [60, 73]	64 [57, 71]	70 [60, 79]	72 [61, 83]	60 [49, 72]	61 [53, 66]†	62 [54, 69]	70 [58, 81]
MAP, mmHg	91 [84, 97]	78 [72, 84]	85 [78, 91]	82 [75, 88]	87 [78, 96]	89 [79, 100]	76 [64, 89]	78 [69, 88]	79 [70, 87]	88 [75, 100]
HR, beats/min	72 [68, 76]	70 [66, 74]	70 [67, 73]	67 [63, 70]	69 [63, 75]	70 [61, 79]	69 [61, 76]	67 [60, 74]	66 [60, 73]	66 [59, 74]
Body thermal sensation	5.2 (5–6)	4.9 (4–6)	4.7 (4–6)	4.9 (4–6)	5.0 (4–6)	5.2 (4–6)	5.7 (5–6)*	5.2 (5–6)	5.2 (4–6)	5.4 (4–6)*
Body thermal comfort	1	1	1	1	1	1	1	1	1	1
Affective valence	2.9 (1 to 5)	3.0 (1 to 5)	1.1 (−1 to 4)	2.0 (0 to 4)	2.3 (0 to 4)	1.9 (0 to 4)	2.5 (0 to 5)	2.0 (0 to 5)	2.5 (0 to 5)	2.5 (0 to 5)
CON group										
*T*_rec_, °C	37.2 [37.1, 37.2]	37.1 [37.0, 37.2]	37.1 [37.0, 37.3]	37.1 [37.0, 37.3]	37.1 [36.9, 37.2]	37.1 [37.0, 37.3]	37.0 [36.9, 37.2]	37.1 [37.0, 37.2]	37.1 [37.0, 37.2]	37.0 [36.9, 37.2]
*T*_sk_, °C	33.8 [33.4, 34.2]	34.9 [34.7, 35.1]	34.7 [34.5, 35.0]	34.6 [34.3, 34.9]	34.5 [34.2, 34.9]	33.6 [33.5, 33.8]	34.9 [34.7, 35.1]	34.7 [34.4, 34.9]	34.5 [34.2, 34.9]	34.3 [34.0, 34.6]
${\bar{T}}_{\text{b}}$, °C	36.0 [35.8, 36.1]	36.3 [36.2, 36.4]	36.3 [36.1, 36.4]	36.2 [36.0, 36.4]	36.2 [36.0, 36.3]	35.9 [35.7, 36.0]	36.3 [36.2, 36.4]	36.2 [36.1, 36.3]	36.2 [36.0, 36.3]	36.1 [36.0, 36.2]
$\dot{M}$, W	131 [113, 149]	139 [124, 154]	140 [122, 159]	137 [118, 157]	137 [121, 152]	124 [108, 139]	128 [110, 146]	131 [115, 147]	129 [116, 141]	126 [111, 140]
SAP, mmHg	115 [105, 125]	104 [95, 114]	119 [109, 130]	117 [109, 125]	127 [118, 137]	119 [111, 126]	107 [100, 113]	120 [110, 130]	117 [105, 130]	132 [118, 145]
DAP, mmHg	67 [63, 71]	56 [52, 61]	64 [59, 69]	65 [60, 69]	75 [67, 83]	71 [66, 77]	61 [56, 67]	67 [59, 75]	66 [57, 75]	79 [68, 89]
MAP, mmHg	85 [80, 89]	74 [69, 79]	85 [78, 91]	84 [78, 90]	94 [86, 103]	89 [83, 94]	78 [72, 84]	86 [76, 96]	84 [74, 95]	97 [86, 109]
HR, beats/min	69 [63, 75]	69 [63, 74]	69 [62, 75]	67 [60, 75]	67 [61, 73]	69 [63, 76]	68 [61, 74]	67 [62, 73]	65 [59, 71]	67 [61, 72]
Body thermal sensation	5.1 (4–6)	5.3 (5–6)	4.6 (4–6)	4.6 (4–6)	4.8 (4–6)	4.5 (4–6)	5.2 (5–6)	4.7 (4–6)	4.6 (4–6)	4.7 (4–6)
Body thermal comfort	1	1	1	1	1	1	1	1	1	1
Affective valence	2.9 (0 to 5)	2.9 (0 to 5)	1.8 (−1 to 5)	2.4 (0 to 5)	2.4 (0 to 5)	2.9 (0 to 5)	2.8 (0 to 5)	1.9 (−1 to 5)	2.3 (−1 to 5)	2.6 (0 to 5)

Data are the mean of the entire phase. Values are mean [95% confidence intervals] for rectal (*T*_rec_), skin (*T*_sk_) and mean body (${\bar{T}}_{\text{b}}$) temperatures, metabolic heat production ($\dot{M}$), systolic (SAP), diastolic (DAP), and mean (MAP) arterial pressures, and heart rate (HR). Values are mean (range) for thermal sensation and comfort, and affective valence. Physiological data were analyzed with two-way repeated-measures ANOVA, followed by Duncan’s multiple range test. Perceptual data were analyzed with Kruskal–Wallis’ test, followed by either Mann–Whitney *U* or Wilcoxon signed-rank test (*P* ≤ 0.05). B-WI, body water-immersion; B-RW, 30-min body-rewarming; CA group, cold-acclimation group (*n* = 7 men); CON group, control group (*n* = 7 men). H-CWI, 30-min hand cold-water immersion; H-RW, 15-min hand-rewarming.

Significantly different from the preacclimation trial (*) and from the CON group (†).

The right-finger CVC was similar in all trials, both in the baseline (CA group: Pre = 3.4 [2.4, 4.5] PU·mmHg^−1^, Post = 3.7 [3.0, 4.5] PU·mmHg^−1^, and CON group: Pre = 3.5 [2.7, 4.3] PU·mmHg^−1^, Post = 3.9 [3.0, 4.8] PU·mmHg^−1^; *P* = 0.78, ${\text{η}}_{\text{p}}^{\text{2}}$ = 0.006) and the B-WI (CA group: Pre = 4.0 [3.0, 5.1] PU·mmHg^−1^, Post = 4.6 [3.6, 5.6] PU·mmHg^−1^, and CON group: Pre = 4.2 [3.3, 5.1] PU·mmHg^−1^, Post = 4.7 [3.8, 5.6] PU·mmHg^−1^; *P* = 0.89, ${\text{η}}_{\text{p}}^{\text{2}}$ = 0.001) phases. The cold-induced reduction in the right-finger CVC did not differ between trials and groups (*P* = 0.88, ${\text{η}}_{\text{p}}^{\text{2}}$ = 0.001; Fig. 5*B*). No intertrial differences were observed in the right-finger CVC, either in the H-RW (*P* = 0.74, ${\text{η}}_{\text{p}}^{\text{2}}$ = 0.009) or in the B-RW (*P* = 0.53, ${\text{η}}_{\text{p}}^{\text{2}}$ = 0.03) phases (Fig. 5*B*).

$\dot{M}$, SAP, MAP, and HR did not differ between trials (*P* > 0.05; Table 4). In the CA group, DAP was lower in the post- than in the pre-H-CWI (*P* = 0.05; Table 4). Also, the magnitude of MAP elevation instigated by the localized cooling was lower in the post-CA than in the pre-CA (*P* = 0.05, *d* = 0.13) and the post-CON (*P* = 0.04, *d* = 0.18) H-CWIs (Fig. 3*B*).

The regional (right hand) thermoperceptual responses obtained during the H-CWI phase are depicted in Fig. 4*B*. In the posttrials, the right-hand thermal discomfort was attenuated in the CA group (*P* = 0.01, *r* = 0.89), but not in the CON group (*P* = 0.36, *r* = 0.34). Also, the regional sensation of coldness was attenuated in the CON group (CA group: *P* = 0.09, *r* = 0.63 and CON group: *P* = 0.04, *r* = 0.76), whereas the cold-induced pain was alleviated in both groups (CA group: *P* = 0.01, *r* = 0.89 and CON group: *P* = 0.04, *r* = 0.74). Furthermore, after the acclimation, the CA group perceived the whole body temperature slightly warmer (*P* = 0.04, *r* = 0.76; Table 4).

## DISCUSSION

The present study tested the hypothesis that a short-term, whole body cold acclimation regimen would improve finger vasoreactivity and thermosensitivity to direct localized cooling. To this end, we used a between-subjects design, wherein seven healthy men (i.e., CA group) encountered, in a controlled manner, repeated reductions in body core (∼1.5°C) and whole body surface (∼11°C) temperature by means of daily static ≤2 h immersion in 14°C water over a 5-day period, whereas seven other men (i.e., CON group), with similar physical characteristics as those in the CA group, avoided any cold exposure. To eliminate any thermal influence from variations in the hand skin temperature on the adaptation process, we utilized a closed-loop acclimation protocol, during which the right-hand finger temperature of the CA group was maintained at ∼35.5°C throughout the 14°C-immersion sessions. Before and after the 5-day intervention, finger vasomotor and thermoperceptual responsiveness to a fixed localized cold stimulus (i.e., a 30-min hand immersion in 8°C water) was evaluated in both groups, and in two different whole body thermal states, i.e., while subjects were mildly hypothermic and normothermic. The experimental conditions were standardized across the testing trials, with regard to the changes in deep body and skin temperature obtained in the respective thermal condition, the pre-immersion thermal state of the fingers, the immersion duration, the body posture, and the timing of testing.

In the CON group, finger temperature and CVC did not differ between testing periods, and, if anything, the cold-induced drop was slightly aggravated during the post-acclimation cold-provocation trials. In the CA group on the contrary, the 5-day acclimation regimen appeared to attenuate finger constrictor responsiveness to cold, as indicated by the elevated finger skin temperature and the increased number of CIVD episodes. Notably, these regional thermoregulatory adjustments, which were restricted to the local cooling (i.e., H-CWI) phase of the trials, were prevalent not only in the normothermic conditions but also when the subjects were rendered mildly hypothermic, hence overriding the powerful constrictor influence of the deep-body cooling (6, 32–34). Present findings , therefore, support our hypothesis, that, in healthy men, repeated whole body exposure to severe cold may evoke peripheral thermal adaptations.

During a cold challenge, cutaneous vasomotion is governed by complex and dynamic interactions of whole body and local influences, regulated through both neural and nonneural mechanisms (39, 40). In the present study, the regional thermoadaptations obtained in the CA group cannot be ascribed to local changes occurring at the level of finger-skin thermoreceptors, given that, during the acclimation process, no direct thermal stimulation was applied to the right (test) hand. Rather, the diminished constrictor response in finger arterioles was probably mediated wholly by acclimation-evoked alterations in central circuits, modifying the reflex neural control of acral-skin vasomotion. The acclimation regimen did blunt the magnitude of the systemic pressor reactivity to localized cold stress, reflecting the induction of central nervous habituation, which presumably was associated with a lower increase in sympathetic outflow directed to the acral-skin vasculature during the local cold stimulation. The current experimental design does obviously not allow us to delineate which region(s) along the sympathetic reflex axis was (were) affected by the repetitive whole body cold stimuli. A generalized sympathetic hypo-activation to acute cold stress has indeed been indicated by several cold-acclimation studies (cf., inter alia Refs. 41–46). For instance, Makinen et al. (47) have demonstrated that, after 10 consecutive days of a 2-h exposure to 10°C air, the autonomic balance during acute cold was shifted toward decreased sympathetic and increased parasympathetic nerve activity. Furthermore, the cold-evoked elevation of circulating catecholamines, especially of norepinephrine, is often attenuated following prolonged periods of intermittent body cooling (47–49).

Arguably, the 5-day cold acclimation might also have altered the systemic concentration of other metabolic (e.g., nitric oxide—a potent vasodilator) and/or hormonal (e.g., endothelin-1—a potent vasoconstrictor) regulators of cutaneous circulation. For example, in rats, a week of sustained exposure to cold enhanced endothelin-1 concentration in certain cardiovascular structures (50). It should nevertheless be noted that palmar-finger skin circulation is predominantly regulated by the noradrenergic vasoconstrictor system (39), and thus it seems unlikely that any potential changes in endothelin-1 and/or nitric oxide might have contributed to the finger vasomotor responses observed in this study (51, 52). Moreover, to our knowledge, the impact of whole body cold adaptation on endothelial function has as yet not been examined in humans (see Ref. 53), whereas a 2-wk local (hand) cold-acclimation protocol failed to modulate the plasma levels of endothelin-1 and nitric oxide (18).

During the posttrials, the noxious (8°C) thermal stimulus was perceived less painful (in the mild-hypothermic and normothermic trials) and not as cold (in the normothermic trial). These responses apparently were not triggered by the cold-acclimation intervention, because they prevailed in both groups alike. On the basis of previous work showing that nociceptive habituation develops relatively promptly (22, 54, 55), we assume that the modified sensations may describe a rapid thermoperceptual adaptation that was driven by the initial (pretrials) local cold provocations. That the thermoperceptual output may have been influenced by subjects’ familiarization with the experimental procedure (i.e., placebo effect) constitutes an alternative explanation. In any event, the universal mitigation of cold-induced pain postacclimation suggests that the diminished autonomic reactivity in the CA group was independent of the changes in pain perception (56, 57).

Contrary to the discriminative and nociceptive responses, the cold acclimation regimen partly alleviated the hedonic perceptions to localized cooling; that is, in the posttrials, the 8°C water was assessed less thermally uncomfortable only by the CA group. Although speculative, such a selective diminution of the temperature-related sensations might be attributable to the differential influence of the repetitive whole body cold stress on specific brain regions that are responsible for the sensory and affective attributes of local thermal stimuli. Thus, the primary area for thermal sensation is the insular cortex, whereas thermal pleasantness is associated with activations also in the orbitofrontal and cingulate cortices (58–60). Furthermore, it is plausible that the blunted regional discomfort was driven largely by the more positive generalized affective state, altering the central integration and processing of thermo-sensory information (61). In this regard, it is of interest that emotions also may impact the acral-skin vasomotor responses to cold (62).

### On Whole Body Cold Adaptation

During the acclimation process, a *specific* adaptation to severe (14°C) cold stress manifested itself: metabolic (i.e., lower cold-induced elevation in $\dot{M}$, as well as in perceived shivering), cardiovascular (i.e., lower HR), and thermoperceptual (i.e., attenuated sensation of coldness, displeasure, and pain) responsiveness to whole body cold stimulus were blunted. These thermoregulatory adjustments, typically attained after repeated exposures to low ambient temperature (30), did not occur simultaneously but developed at different time points of the acclimation process. That is, prominent reductions in the objective and subjective indexes of shivering intensity, as well as in HR and thermal pain, were detected already at the second acclimation session, whereas the self-reported alleviation of thermal discomfort and coldness emerged during the last two sessions. Previous findings on the time-course of the cold-evoked thermoeffector adaptation are inconsistent; yet the prevailing view is that thermoperceptual adjustments precede any changes in the autonomic responses (29, 30).

Notably, the majority of the whole body thermo-adaptations evoked by repeated exposure to severe (14°C) cold were *transferred* to moderate (21°C) cold-stress exposure: subjects shivered less, and perceived the 21°C water less cold and thermally uncomfortable after acclimation. Surprisingly, a similar drop in shivering thermogenesis was observed also in the CON group, who deliberately avoided any exposure to cold throughout the intervention period. The mechanism behind the metabolic downregulation in the CON group is not clear. Yet the findings appear similar to that by Tipton et al. (46), who observed, in seven nonacclimatized men (control group), that the cold-induced elevation in O_2_ uptake was ∼10%–15% lower (albeit not statistically significant; see Fig. 1 in Ref. 46) in the second than in the first 12°C-water immersion, performed 1–2 wk after the first immersion.

During the normothermic trial, subjects always perceived the 35.5°C water thermally comfortable. Yet, after the cold acclimation, six of seven CA subjects judged the overall thermal stimulus slightly warmer, thus indicating a small shift of the perceptual thermoneutrality toward a lower ambient (water) temperature. This alliesthetic response was independent of any intertrial variation in the temperature of the water (Pre: 35.4°C vs. Post: 35.3°C; *P* = 0.66), as well of the subjects’ core and surface temperature (Table 4). Moreover, the altered temperature sensation was not accompanied by any changes in autonomic thermoeffector responses: CVC and $\dot{M}$ did not differ (Table 4), and none of the subjects self-reported upperbody wettedness, suggesting sweating, across the trials. Regardless of the underlying mechanism, the finding of changed thermal sensation at given body temperatures seems to be consistent with the notion that the “thermoneutral zone” and the “thermal comfort zone” are distinguishable (63).

### Methodological Considerations

Subjects were requested to avoid any severe cold exposure (e.g., skiing, cold baths, etc.), and to maintain, when necessary, a thermally comfortable microenvironment (e.g., wearing warm clothes, gloves, etc.) throughout the study period. During the postacclimation visits, subjects verbally confirmed their compliance to these guidelines, yet no direct assessment of their habitual outdoor activities was performed. The study would also have benefited from the inclusion of an additional control group that would have been immersed daily in thermoneutral water. We did not include women in this study due to the influence of the female reproductive hormones on autonomic thermoregulation (64) and thermal perception (65). Future work however is required to determine whether, or to what extent, the impact of cold acclimation on cutaneous vasomotion and thermoperception is sex dependent. Despite the relatively homogeneous group of subjects tested (as regards age, sex, health status, and morphological characteristics) and the strictly controlled acclimation protocol [as regards the total thermal-stress volume (i.e., intensity × duration × frequency of cold stimulus applied)], an interindividual variability on the direction and the magnitude of the thermo-adaptative response was evident. Yet the present experimental design does not allow us to identify the potential source(s) of this variation. It should also be highlighted that our observations are limited to the acral-skin vasculature of the hand, and thus cannot be extrapolated to the nonacral skin sites, nor to acral sites of the feet; especially in view of previous work indicating that the thermo-adaptive capacity of the upper and lower extremities may differ (22, 66). Finally, it remains to be settled if the finger vasomotor reactions obtained during cold-water immersion would be similar to those during cold-air exposure.

### Perspectives and Significance

In view of the premise that the CIVD response might have a cryoprotective function (3, 4), it is reasonable to assume that the finger thermoregulatory responses attained after the whole body cold acclimation, might decrease susceptibility to frostbite. Still, whether the magnitude of thermo-adaptations induced by the present acclimation regimen would be sufficient to reduce the risk of developing local cold injury, and/or to improve manual performance in cold environments, remains unknown.

In conclusion, present findings demonstrate that, in healthy individuals, perturbations of thermal homeostasis imposed by repeated whole body exposures to severe cold during the course of a week may attenuate finger vasoreactivity and thermosensitivity to direct localized cooling. These regional thermo-adaptative modifications appear to be mediated by central nervous habituation induced by the iterative, generalized cold stimulation. Future studies are warranted to investigate the exact neural mechanisms underlying the thermoregulatory plasticity of acral skin in response to prolonged intermittent deep-body and/or skin-surface cooling.

## GRANTS

The study was funded from the Swedish Armed Forces Grant No. 92220918; M. E. Keramidas was supported by a salary grant from the Kungliga Tekniska Högskolan (KTH)-Royal Institute of Technology under Grant No. 65276.

## DISCLOSURES

No conflicts of interest, financial or otherwise, are declared by the authors.

## AUTHOR CONTRIBUTIONS

M.E.K. and O.E. conceived and designed research; M.E.K., R.K., P.G., F.W., and A.E. performed experiments; M.E.K. analyzed data; M.E.K. and O.E. interpreted results of experiments; M.E.K. prepared figures; M.E.K. drafted manuscript; M.E.K. and O.E. edited and revised manuscript; M.E.K., R.K., P.G., F.W., A.E., and O.E. approved final version of manuscript.
